# Understanding and Imitating Unfamiliar Actions: Distinct Underlying Mechanisms

**DOI:** 10.1371/journal.pone.0046939

**Published:** 2012-10-10

**Authors:** Joana C. Carmo, Raffaella I. Rumiati, Antonino Vallesi

**Affiliations:** Sector of Cognitive Neuroscience, Scuola Internazionale Superiore di Studi Avanzati (SISSA), Trieste, Italy; Weill Cornell Medical College, United States of America

## Abstract

The human “mirror neuron system” has been proposed to be the neural substrate that underlies understanding and, possibly, imitating actions. However, since the brain activity with mirror properties seems insufficient to provide a good description for imitation of actions outside one’s own repertoire, the existence of supplementary processes has been proposed. Moreover, it is unclear whether action observation requires the same neural mechanisms as the explicit access to their meaning. The aim of this study was two-fold as we investigated whether action observation requires different processes depending on 1) whether the ultimate goal is to imitate or understand the presented actions and 2) whether the to-be-imitated actions are familiar or unfamiliar to the subject. Participants were presented with both meaningful familiar actions and meaningless unfamiliar actions that they had to either imitate or discriminate later. Event-related Potentials were used as differences in brain activity could have been masked by the use of other techniques with lower temporal resolution. In the imitation task, a sustained left frontal negativity was more pronounced for meaningless actions than for meaningful ones, starting from an early time-window. Conversely, observing unfamiliar versus familiar actions with the intention of discriminating them led to marked differences over right centro-posterior scalp regions, in both middle and latest time-windows. These findings suggest that action imitation and action understanding may be sustained by dissociable mechanisms: while imitation of unfamiliar actions activates left frontal processes, that are likely to be related to learning mechanisms, action understanding involves dedicated operations which probably require right posterior regions, consistent with their involvement in social interactions.

## Introduction

In recent years, the neural control of imitation has attracted a great deal of attention, with the particular aim of identifying its neural and cognitive mechanisms (see [1] for a review). Both neuropsychological studies (e.g., [2]–[5]) and functional neuroimaging ones [6], [7] suggest that human action imitation is sustained by a fronto-parietal network.

This network, known as the observation-execution matching system, has been proposed to be the human homologue of the monkey’s “mirror neuron system” (MNS) constituted of multimodal neurons in the ventral premotor and inferior parietal cortices that discharge not only when an animal executes a goal-directed gesture involving an object or a tool, but also when it observes or predicts the same gesture [8]–[10]. There are however two clear elements of discontinuity between humans and monkeys: monkeys do not imitate tout court and mirror neurons do not respond to executed or observed meaningless actions (see [11] for a review).

This execution-observation matching system has been identified as the neurophysiological correlate of a direct matching mechanism, whereby an observed action activates the stored motor-related representation [12]. This hypothesised mechanism can also account for imitation of actions providing that they are already present in one’s motor repertoire (familiar actions). For reproducing novel motor patterns, however, a supplementary mechanism, related to activity within the middle frontal gyrus has been proposed [13], [14] that allows observed novel actions to become part of the observer’s own motor repertoire [15].

Previous studies investigating the brain mechanisms subserving imitation of familiar and unfamiliar gestures differ greatly for the experimental procedures they adopted, thus making the comparison between them troublesome. Some used unfamiliar gestures [13], [16], familiar actions [17] or simple pre-determined finger movements [6]. Other studies employed both familiar and unfamiliar actions favouring the observation of both commonalities and differences in brain activity. In some of these studies, activations associated with familiar and unfamiliar actions were obtained either by contrasting the task against a baseline condition [18], [19], or by comparing familiar and unfamiliar actions against each other [7], [14], [20], [21].

The main activations in these studies were the following. The premotor cortex (SMA, BA6) has been found to be recruited when individuals imitate either familiar or unfamiliar actions across different studies [7], [13], [19], [21]; the recruitment of the superior parietal cortex has been observed when individuals imitate both familiar actions [19], [21] and unfamiliar actions (e.g., [7], [13], [18,]), while the inferior frontal cortex (ventral premotor, BA44), bilaterally or in the left hemisphere, has been reported to play a role during imitation of unfamiliar gestures (e.g., [14], [16], [22]), or with pre-instructed finger movements [6], but only once with familiar actions [17]. Notwithstanding that imitation of both familiar and unfamiliar actions does recruit a common network, the participation of the orbito-frontal areas (BA11) has been found for imitation of familiar actions only (e.g., [14], [19]). Moreover, two studies in which participants were asked to reproduce more complex unfamiliar actions (i.e., playing the guitar) reported activations in the middle frontal gyrus (BA9, BA46) [13], [14], areas that the authors suggested to be specifically related to imitation learning mechanisms.

Observation of actions recruits the same neural structures that are also involved in the actual execution of the observed actions (e.g., [19], [23]–[26]). Thus, observing actions for later execution (vision for action) or simply watching them (vision for perception) did not lead to marked differences in brain activity with parietal and premotor regions being recruited in both cases [19], [27]. However, when the intention to observe actions had the specific goal of recognizing the viewed action (vision for recognition), the observed activity was along the ventral visual pathway [27], [28] (see also [29]). Additional activation in the right superior temporal sulcus (STS) when participants observed hand actions has also been reported [30], [31], supporting its key role in action understanding [32].

Despite the general interest in imitation, not much investigation has been carried out to date on the time course in brain activation of the processes involved in action observation and action imitation except for a magnetoencephalographic study (MEG) [22] and one in which the Event-Related Potential (ERP) methodology was employed [33]. The main findings in Nishitani and Hari [22] were first an activation observed in the left occipital areas, then in the left inferior frontal cortex (BA44), followed 100–150 msec later by an activation in the left sensorimotor cortex (BA4) and only 100–200 msec later by an activation in the right BA4. The fact that the left BA44 was activated significantly earlier than the left BA4 area in all conditions led the authors to suggest a role of the BA44 as an orchestrator for the observation-execution system.

ERPs have been used to study both imitation [33] and observation (e.g. [34]). While imitation was associated with an early activation around the motor areas [33], action understanding was associated with an N400-like potential within a semantic network when participants observed meaningless as compared to meaningful hand actions [34]. Recently, Ortigue and colleagues [35] measured Visual Evoked Potentials as they investigated the temporal sequence of brain topographies (and their sources) when participants observe familiar and unfamiliar actions. This study shows 4 main steps when participants observe hand-object interactions: a bilateral posterior activity; a successive strong activation of left posterior temporal and inferior parietal cortices; an increase in activations of the right temporal parietal cortices that differ significantly by action-type; and a bilateral orbito-frontal activation. The early left activations (<200 ms) were interpreted as being part of a lateralized observation-execution network and the successive right activation (aprox. 300 ms) as playing a role in understanding the intentions of others and agency.

By using ERPs in the current study which adopted a full factorial design, we aimed at investigating the differences in the neural mechanisms underlying participants’ observation of familiar and unfamiliar actions with the intention to either imitate or discriminate them. On the one hand, this design allowed us to ascertain whether imitation of unfamiliar actions is accomplished by the same functional mechanisms engaged when imitating familiar actions or by different ones. The latter possibility is suggested by the studies showing that the areas with mirror properties seem insufficient to account for imitation of gestures that are not yet part of one’s own repertoire [13], [14]. On the other hand, we aimed at evaluating whether discriminating familiar and unfamiliar actions lead to marked differences and whether the electrophysiological pattern of activity would be compatible with a typical fronto-parietal network, a ventral visual pathway, or both. Finally, if both imitation and understanding of actions are accomplished by the same underlying cognitive processes, and given that the same actions are presented to all participants, no dissociation should be found either in the ERP time-course or in the scalp topography.

## Methods

### Ethics Statement

This study was previously approved by SISSA ethics committee.

### Participants

Eighteen right-handed Italian adults participated in the study (13 females; mean age = 22.8, SD = 2.2 years). Handedness was assessed with the Edinburgh Handedness Inventory (mean = 84, SD = 20). All participants had normal or corrected-to-normal visual acuity. Participants gave written informed consent and received 20 Euros for their time.

### Stimuli and Design

The stimuli used in both tasks comprised 12 meaningful (MF) and 12 meaningless (ML) hand gestures (for a total of 24 different gestures). The video-clips lasted 2000 msec each and were performed by a male model. The set of MF actions comprised 12 object-unrelated, symbolic gestures (e.g., wave goodbye, see Supporting Information S1 for a complete list), while the set of unfamiliar ML actions was obtained by modifying the relationship between hand–arm and trunk of each of the 12 MF actions (a detailed description of how ML actions have been obtained can be found in [36].

The experiment was conducted in a sound-attenuated room. Each trial started with a fixation cross that lasted for 2000 msec. After this period, the video-clip was presented for 2000 msec followed by a 500 msec blank, at the end of which the response slide was presented until the participants’ response. During the video presentation, for either the discrimination or imitation task, participants were asked to keep one button pressed with their right index finger. The requirement to press a button during video presentation (and EEG recording) was introduced to prevent participants from beginning to imitate before the critical time-window for ERPs, as movement artefacts are well known to severely affect EEG traces. Discrimination and imitation tasks differed only for the response slide. In the discrimination task, participants were prompted to answer the question ‘Does it have a meaning?’ (in Italian: ‘Ha un significato?’), and required to press one of two additional buttons labelled ‘Yes’ (‘Si’) and ‘No’. In the imitation task, participants were prompted with ‘Now imitate the gesture’ (‘Ora imita l'azione!’) and were instructed to release the target button and imitate with the same hand as in the observed action. At the beginning of each task session, participants had a practice session of 12 trials, thus they were aware of which task they were going to perform afterwards while observing the video stimuli. Both sets of MF and ML actions were repeated 4 times in each task, for a total of 192 trials. The order of administration of the two different tasks (imitation versus discrimination) was counterbalanced across participants. In the middle of each task, a short pause was given to allow some rest and check electrode offsets. In order to exclude incorrect trials, participants’ performance was video-recorded and scored off-line by one experimenter.

### Electrophysiological Recordings and Data Pre-processing

EEG signal was continuously sampled at 256 Hz using a BioSemi ActiveTwo system (http://www.biosemi.com/) from a pre-cabled cap with 128 pin-type active Ag/AgCl electrodes (with a modified 10–20 system montage). Minimal skin preparation was necessary with active electrodes. Eight additional drop-down electrodes were attached around the participant’s face: horizontal electro-oculographic (EOG) signals were recorded at the left and right external canthi, vertical EOGs were recorded below the eyes, and 4 additional electrodes were placed bilaterally on the mastoids and peri-auricular sites. Individual electrodes were adjusted until the electrode offset was within the range of ±40 mV.

Pre-processing of the EEG data was done in EEGLab v7.2.9.19b [37]. All electrodes were referenced offline to the average-reference. Continuous EEG signal was high-pass filtered at 0.1 Hz and low-pass filtered at 30 Hz to remove high-frequency noise produced by muscle-related activity and external electrical sources. Continuous EEG was segmented into epochs from 400 msec before video onset to 2000 msec after, for correctly performed trials only. On average, correct trials were 93.68% (SD = 3.89) for imitation, and 90.61% (SD = 4.93) for discrimination. Channels with amplitude exceeding the threshold of +/-150 µV in more than 30% of the trials were interpolated. An automatic epoch rejection algorithm was used, with threshold set to -/+1000 µV and allowing a maximum of 5% of rejections per iteration. In order to detect and remove eye-artifacts, the EEG signal was decomposed using Independent Component Analysis (as implemented in EEGLab), and independent components attributed to blinks and eye-movements after visual inspection were eliminated from the data. For each condition, before ERP averaging, we further excluded from single epochs the electrodes in which more than 10 time-points (out of 614) overcame the threshold of +/-100 µV. All ERPs were baseline-corrected using the 400 msec pre-video period. For the ERP analyses, on average, there were 36.2 good trials (SD = 5) in the discrimination task and 37.2 good trials (SD = 3.7) in the imitation task.

### ERP Data Analysis

**Figure 1:**
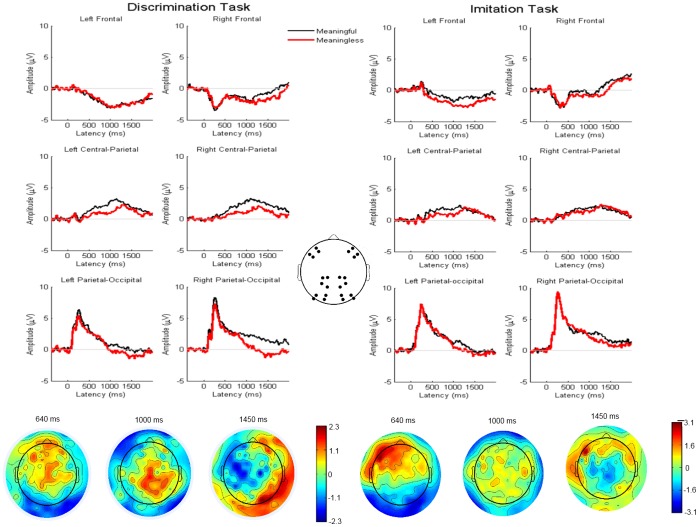
Grand average ERPs and Topographic amplitude difference for meaningful and meaningless actions. Grand average ERPs from imitation task (right panel) and discrimination task (left pannel) for meaningful and meaningless actions, for the frontal, centro-parietal and parietal-occipital ROIs. Central panel: schematic illustration of location of electrodes that each ROI comprises. At the botton: topographic distribution of amplitude difference between meaningful and meaningless conditions including all scalp sites for discrimination (left) and imitation tasks (right), at 640 ms, 1000 ms, and 1450 msec.

Across all participants, we assessed the difference in the mean amplitudes of the ERPs from MF and ML trials, for both imitation and discrimination tasks. ERPs analysed were those recorded during action observation only, to minimize movement-related artefacts. Based on visual inspection and topographic activity distribution for both task conditions, we chose three scalp regions of interest (ROIs) that captured the most prominent effects observed in our study, in order to maximize variations in the underlying cognitive processes of both tasks. Each ROI encompasses the electrode that shows the maximum MF versus ML difference in a given task and the three closest neighbouring electrodes (see Fig. 1). For each ROI, a time-window of 150 msec was chosen and centered at the time point of MF and ML maximum difference in amplitude. For each ROI we chose their homologous counterpart in the opposite hemisphere, that served as control ROIs. The choice of these control regions was made a priori, and is therefore unbiased.

A clear dipole-like voltage pattern can be identified with negativity at the left frontal sites and positivity at right posterior sites in the ML-MF contrast for the imitation task at the first time-window (565–715 msec). This effect shows the maximum difference between MF and ML conditions at electrode F5’ around 640 msec after video stimuli onset. The left frontal ROI thus comprises the electrodes closest to F5’: AF5’, F7, AF7’ (a standard 10/10 system nomenclature is used here instead of the Biosemi one, to favour the comparison with the literature). The right counterpart is the region that includes electrodes F6’, AF6’, F8, AF8’.

At a middle time-window (925–1075 msec), a central-parietal right-lateralized positivity was identifiable for the discrimination task (see Fig. 1). The electrode CP2’ corresponds to the place where this effect reaches its maximum in amplitude differences, and this occurs at 1000 msec after stimulus onset. This central parietal region comprises the electrodes CP2, P2, CP2’ and P2’ in the right hemisphere, while its left counterpart included electrodes CP1, P1, CP1’ and P1’.

For the discrimination task, a final component with negativity over right parieto-occipital scalp regions was observed much later (1475–1525 msec). To explore this effect, we selected the PO4’ electrode with maximum difference between MF and ML actions from inspection of the topographic map. The time-window for this effect was centered at 1450 msec after stimulus onset, the sample point where the difference reaches its maximum at PO4’ electrode. The right parieto-occipital region comprises PO4’ and its closest neighbouring electrodes: PO2’, PO6’, PO8. The left counterpart included electrodes PO1’, PO3’, PO5’, PO7.

For each time-window, a repeated-measures ANOVA was performed on the mean amplitude values of correct trials for each participant, with Task (Discrimination, Imitation), Meaning (Meaningful, Meaningless), ROI (Frontal, central-parietal, parieto-occipital) and Lateralization (Left hemisphere, Right hemisphere) as independent variables. Post-hoc Tukey’s Honestly Significant Difference Test was used to detect the sources of the effects involving more than two conditions.

## Results

### Left Frontal Component Activity (565–715 msec)

At this time-window, a broadly distributed negativity was observed over the most anterior region with its inverted counterpart towards the most posterior regions (main effect of ROI: *F*(2,34) = 22.5, *p*<.0001). Although we observed a mean amplitude potential lower for the right hemisphere than for the left hemisphere (main effect of hemisphere: *F*(1, 17) = 16.1, *p*<.001), we observed a significant hemisphere × ROI (*F*(2,34) = 5.1, *p* = .011) interaction, with this effect being present at the frontal sites only (Tukey *p*<.001), and absent at the central parietal (Tukey *p* = .99), and at the parieto-occipital (Tukey *p* = .31) scalp sites. Overall ML actions had a lower mean amplitude than MF actions (Main effect of meaning: *F*(1, 17) = 6.6, *p* = .01), while this was not the case for the most posterior scalp region (Tukey *p* = .88), where this effect was slightly reverted (Meaning × ROI interaction: *F*(2, 34) = 4.1, *p* = .025).

Critically, a significant Task × Meaning × ROI × Hemisphere interaction (*F*(2, 34) = 3.97, *p* = .028) was found at this time-window. Based on subsequent separate ANOVAs for each of the three ROIs, we were able to detect that the difference between imitation and discrimination task was present mainly at the left frontal region (Task × Meaning: *F*(1, 17) = 8.87, *p* = .008); Task × Meaning × Hemisphere: *F*(1, 17) = 15.80, *p* = .001), while it did not occur in the Central-Parietal ROI (Task × Meaning: *F*(1, 17) = 1.78, *p*>.1; Task × Meaning × Hemisphere: *F*(1,17) = 1.79, *p*>.2), and it was reversed in the Parieto-occipital ROI (Task × Meaning: *F*(1, 17) = 2.56, *p*>.1; Task × Meaning × Hemisphere: *F*(1,17) = 6.22, *p* = .02).

**Figure 2:**
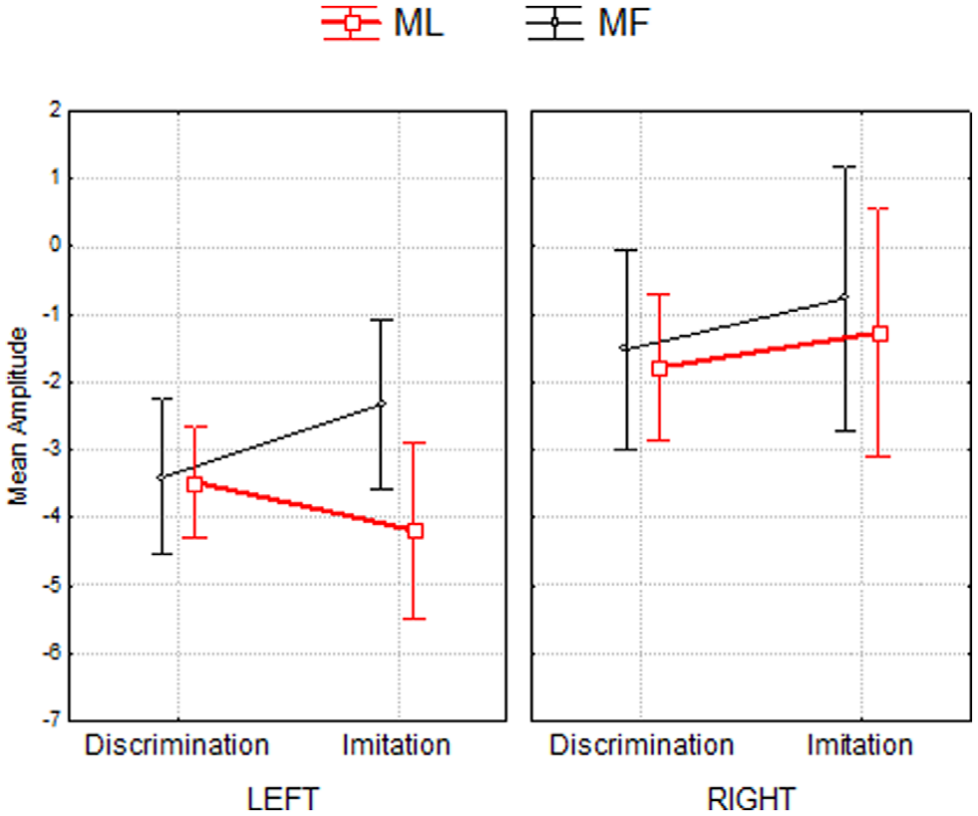
First time window (565–715 ms). Mean Amplitude from imitation and discrimination tasks, MF and ML actions for the frontal ROIs at the first time window (565–715 ms). Error bars denote 95% confidence intervals.

Post-hoc comparisons further confirmed that this 3-way interaction found at the frontal ROI (Fig. 2) is driven by greater negativity for ML actions as compared to MF actions at the left hemisphere in the imitation task (Tukey *p*<.001); while the difference between MF and ML actions is not present in the discrimination task (Tukey *p* = .99). The 3-way interaction found at the parieto-occipital ROI is driven mostly by the absence of differences between the 2 hemispheres for ML action in the imitation task (Tukey *p* = .09), as overall the right hemisphere leads to a higher mean amplitude than the left hemisphere.

### Right Centro-Parietal Component (925–1075 msec)

**Figure 3:**
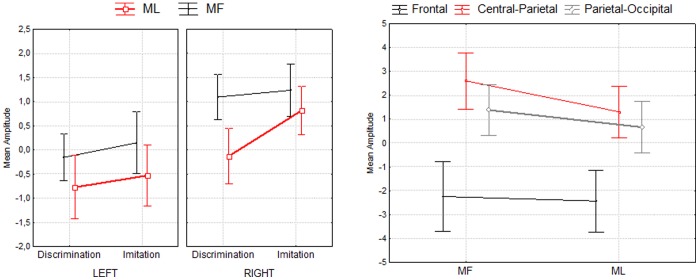
Second time window (925–1075 ms). Mean amplitude of MF and ML actions at the middle time window (925–1075 ms). The right plot depicts overall mean amplitude of MF and ML actions for each of the 3 ROIs. The left plot depicts mean amplitude of MF and ML actions for each task (imitation, discrimination) in the left and right hemisphere. Error bars denote 95% confidence intervals.

At this time-window overall MF actions show a higher mean amplitude than ML actions (main effect of Meaning: *F*(1,17) = 25, *p*<.001). The meaning × ROI interaction (*F*(2,34) = 4.4, *p* = .02, see Fig. 3), suggests that not all regions are contributing equally. Indeed, a higher amplitude of MF actions as compared to ML actions is only visible in the central-parietal region (Tukey *p*<.001), while MF and ML actions did not differ significantly in the frontal region (Tukey *p* = .97) and parieto-occipital ones (Tukey *p* = .09).

Moreover, a Task × Meaning × Hemisphere interaction was found (*F*(1,17) = 11, *p* = .004, see Fig. 3), suggesting that the difference between MF and ML actions found previously in the central-parietal region differed across tasks and across hemispheres. Although at the left hemisphere MF and ML actions differ both for the Discrimination task (Tukey *p* = .003) and the Imitation task (Tukey *p* = .001), at the right hemisphere this difference was even enhanced for the discrimination task (Tukey *p* = .0001) but tended to disappear for the imitation task (Tukey *p* = .071).

### Right Parieto-occipital Component (1475–1525 msec)

**Figure 4:**
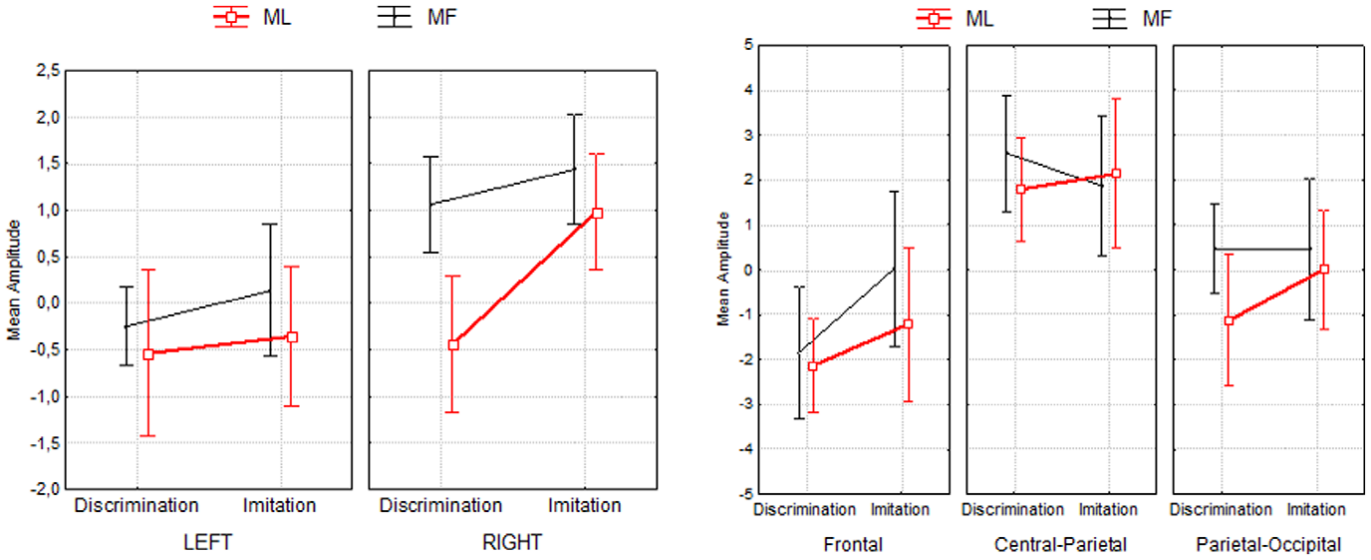
Third time window (1475–1525 ms). Mean amplitude of MF and ML actions for both tasks (imitation, discrimination) at the latest (1475–1525 ms) time window, for each hemisphere (left panels) and for each ROI (right panels). Error bars denote 95% confidence intervals.

At the latest time-window a Task × Meaning × Hemisphere interaction was found (*F*(1,17) = 11.76, *p* = .003), with the difference between MF and ML actions being present for the discrimination task in the right hemisphere (Tukey *p*<.001) but not in the left hemisphere (Tukey *p* = .7) (see Fig. 4). In the case of the Imitation task, no differences between MF and ML actions were found in either hemisphere (for all, Tukey *p*>.15).

The difference between MF and ML actions found in the discrimination task in the right hemisphere is supported by a Task × Meaning × ROI interaction (*F*(2,34) = 4.49, *p* = .01) in which, for the discrimination task, Meaning did not differ in the most anterior region or in the central region (for all, Tukey *p*>.5) but in the parieto-occipital region only (for all, Tukey *p* = .01). For the imitation task, no significant differences between MF and ML actions were found (for all, Tukey *p*>.1).

### Discussion

In this study, Event-related Potentials were recorded upon observation of familiar or unfamiliar communicative gestures, with the same video stimuli being used when participants observed actions with the intention both to imitate and to decide about the meaning of the same actions. A key result was a sustained negative component with a first maximum at around 640 msec over left anterior scalp sites. This frontal component was more pronounced for unfamiliar meaningless actions, when the intention behind action observation was that of subsequent imitation. The fact that this component was less pronounced for familiar actions and that this difference was absent when participants were not required to learn actions for later reproduction (i.e., in the discrimination task), suggests that it underlies imitation learning mechanisms. In imitating unfamiliar actions, a translation between the visual input and motor output is needed [38], as no representation can be retrieved from memory. Hence, this learning mechanism is plausibly more dependent on working memory and under supervisory control of the left dorsolateral prefrontal cortex, as already proposed by Vogt and colleagues [14].

Moreover, according to the Hemispheric Encoding/Retrieval Asymmetry (HERA) model [39], the left frontal lobe is critical for encoding novel aspects of incoming information. Novelty of the incoming information plays a central role, with elaborate encoding processes being applied as a function of it, thus determining long-term storage likelihood [40], [41]. Critically, slow negative potentials recorded from scalp sites over the left dorsolateral frontal sites have been reported to strongly correlate with the level of associative learning [42]. The idea that MNS representations need not be innate and that general learning processes, by concurrent experience of observing and executing actions, could account for their development [43] is not at odds with our claim that learning mechanisms are recruited in imitating unfamiliar actions. These authors propose that factors like contingency and contiguity of the observed and executed actions enable the organisms to build through development, and experience with the environment, common coding representations that help in subsequent imitation and interaction with others [43]. It is our belief that the left frontal component found when one has to contingently imitate an observed action, which is more pronounced for to-be-imitated actions that do not yet belong to one’s own motor repertoire, reflect the requirement for a general learning mechanism.

This frontal activity is also consistent with the idea of a left-lateralized and hand-independent network for planning and execution of both transitive and intransitive gestures, that comprises not only parietal regions but also temporal and frontal ones (e.g., [44], [45]). Having tested action execution of the participants' right limb, we cannot exclude the possibility that this factor may partially contribute to the observed left lateralization. Nonetheless, both neuropsychological studies [2], [3], [5], [46] and neuroimaging studies [7], [44], [45], [47] on praxis brain-related network have provided consistent data that make the interpretation of the left-frontal potential as due to the preparation of right-hand movements only highly unlikely. For instance, Haaland et al. [46] reported that, regardless of hand use, apraxic patients show maximal lesion overlap within the left middle frontal gyrus and/or premotor cortex, and regions around the intraparietal sulcus. Moreover, evidence from neuroimaging shows that a left fronto-parietal network is engaged when advanced planning is required to perform complex motor sequences, regardless of the performing hand [47]. Moreover, the electrode with the maximal ML-MF difference in the early time-window for the imitation task (F5’) is located much more anteriorly than those typically found in ERP investigations of premotor-related planning of volitional movements (e.g., [48]).

The early recruitment of left lateralized frontal processes does not contradict the findings of Nishitani and Hari [22] where the activation of the sensory-motor cortex takes place only after that of the left BA 44. Unfortunately, it is difficult to make a more throughout comparison of the time-course of events between our study and that of Nishitani and Hari [22] due to differences in the methodologies used. While in their study MEG data were time-locked to the endpoint of the modelled action (i.e. pinch of a manipulandum by the experimenter), in ours the brain activity is time-locked to the onset of the modelled action. Moreover, in our experiment, participants performed both tasks at the offset of the modelled action, whereas in Nishitani and Hari’s [22], participants performed an online imitation task, thus being allowed to begin to imitate before the presented action came to an end. Consistently with our results, Vogt et al. [14] showed that the engagement of the left middle frontal gyrus occurs at observation and preparation stages of action production, but not at the execution phase.

A dissociation between imitating and observing actions was also found both at a middle time window (around 1000 msec) and at the latest one (1550 msec). Earlier over right central-parietal and later on over right parieto-occipital scalp sites, an increased negativity was found for unfamiliar meaningless actions when participants prepared to performed the discrimination task. The specific participation of a right lateralized occipito-parietal network has been already reported for both imitation and perception of meaningless actions, as compared to meaningful ones [20]. Decety and colleagues [20] suggest that this finding is probably due to the more complex and demanding process of visuo-spatial inspection of unfamiliar actions. In particular, the right superior-posterior parietal cortex is presumably involved in processing novel translations between visuo-spatial information and motor or body-related information [49]. However our findings are inconsistent with this interpretation, given that an equally substantial, or even higher, participation of the right superior parietal cortex would be expected during observation of unfamiliar action in imitation, which was not the case in our study. Moreover, important differences in the methodologies employed might have in fact proven fruitful in scrutinizing variations in brain activity that might have been obscured by the conjunction analysis performed in Decety and colleagues’ PET study [20].

Alternatively, this right lateralized component over parieto-occipital scalp regions could be partially driven by activity within the Superior Temporal Sulcus (STS). This structure has been implicated in visual perception of biological motion, as it is selectively active for biological-like motion as opposed to non-biological, random motion [50], [51]. Although right, left or bilateral activations of the STS have been observed regarding perception of biological motion [51], it is likely that these differences in laterality are related to the type of stimuli used, as left activations have been shown for object-related actions and right activations for intransitive actions or fine-grained gesturing [30], such as those used in the current study.

Besides the general role played by the STS in perception of biological motion, two studies have shown that this region is strongly engaged when participants observe an incorrect eye gaze movement [50] or grasping movement [52] that violates the observer’s expectancies (goal directed action versus non-goal directed action). These findings led the authors to propose that this structure plays a critical role in social perception, and particularly in the analysis of the intentions of others [50], [52], [53]. Likewise, in our study, it could be the case that when participants observed ML actions, as compared with familiar gestures, their apparent lack of a goal led to a violation of observer’s expectancies when the semantic analysis of the action was stressed by the discrimination (versus imitation) task demands.

The notion of common coding and shared action representations raises the issues of how self versus others distinction comes about, and specifically which neural mechanisms are engaged in discriminating the representation of one self and those activated by external agents [54], [55]. The temporo-parietal junction (TPJ) within the inferior parietal cortex has been proposed to play a key role in the sense of agency [54], [56], with the specific involvement of the right TPJ being systematically associated with 1^st^ person versus 3^rd^ person spatial transformations and perspective taking tasks [57]. When an action is observed, third-person knowledge is conveyed to the observer. Self-generated actions, by contrast, carry first-person types of information, as for instance proprioceptive signals, and it is the presence or absence of the latter signal that would allow a sense of agency [58]. Accumulating evidence suggests that the right TPJ plays a critical role in comparing signals arising from self-produced actions with externally generated actions [57]. Regarding our study, it is possible that, when no representation is available and no imitation processes are operating, as in the observation of unfamiliar actions condition (for later discrimination), there is no need for 1^st^ person versus 3^rd^ person spatial transformations, or less conflict between them, thus explaining the reduction of sustained positivity in the discrimination task for ML actions.

Ortigue and colleagues’ study [35] was, like ours, time-locked to the onset of the observed action. The authors found an early left hemisphere dominance regardless the familiarity of the presented actions followed by the differential recruitment of the right temporal parietal cortices with respect to action type. These findings, despite differences in timing (that could be due to the different complexity and timing of the actions used), are in concordance with ours regarding the discrimination task, as we only found differences between familiar and unfamiliar gestures at latest time-windows which were, like in their study, right lateralized.

In what concerns the relation between these two latest components, we believe that they need not be necessarily related. Nonetheless the fact that the putative engagement of areas that code for agency come only after processes that detect the violation of expectancies by the observation of unfamiliar actions could tell us otherwise. Our tentative interpretation of the latest component that was found for the discrimination of unfamiliar actions is based on the supposed involvement of brain areas that code for the need to distinguish between agents. Thus it could be the case that the STS activity, that we have interpreted as coding for actions that do not yet belong to the motor repertoire and that violate expectancies is feeding the TPJ with this information of the absence of stored motor representations.

In summary, in the present study we show different ERP components during observation of unfamiliar actions, depending on whether participants had to later imitate the presented action or to extract its possible meaning. These components are indicative of distinct supplementary mechanisms recruited during action observation. An early but sustained negative component was found over the anterior left scalp sites when participants prepare to imitate ML unfamiliar actions, compatibly with the engagement of general associative learning mechanisms. On the other hand, an increased negativity was found when participants observed unfamiliar actions (for later discrimination) at a middle time window (around 1000 msec) over the right centro-parietal scalp sites, and later (around 1550 msec) over more posterior sites, suggesting the recruitment of distinct high-order perceptual mechanisms related to the function of right occipito-parietal regions.

In conclusion, based on scalp topography, time course and sensitivity to experimental manipulations, our results suggest dissociable mechanisms for action imitation and action discrimination: imitating unfamiliar actions impose particular demands on a general learning mechanism, while discriminating unfamiliar actions calls for distinct high-order perceptual mechanisms.

## Supporting Information

Appendix S1 **Complete list of meaningful actions used as stimuli.**
(DOC)
