# Supplementary material for: Understanding and Imitating Unfamiliar Actions: Distinct Underlying Mechanisms
Source: PLoS One. 2012 Oct 10;7(10):e46939. doi: 10.1371/journal.pone.0046939 (PMC3468605; doi:10.1371/journal.pone.0046939)
Supplement: Appendix S1 — Complete list of meaningful actions used as stimuli. (DOC) [file pone.0046939.s001.doc]

**Appendix S1**

*Meaningful intransitive actions (for the Italian culture)*

(1) Silence: Closed hand with index finger upright, close to the lips.

(2) Hungry: Straight hand, palm down, hitting repeatedly on the same side of the torso.

(3) Ok: The tips of the index finger and of the thumb make a circle, with the other fingers being open.

(4) More or less: hand open in front of the body; moving repeatedly 45º to the left and to the right (relative to the midline of the body).

(5) Come here: Closed hand, with index finger opening and closing repeatedly.

(6) Good luck: Index finger and middle finger crossed, with all the other fingers closed.

(7) Victory: Index finger and middle finger drawing a V shape.

(8) No: Index finger upright, with the remaining fingers closed, moving 45º to the left and to the right (relative to the midline of the body), repeatedly.

(9) Stop: Open hand, away from and in front of the body.

(10) Bye Bye: Open hand, moving 45º to one side and the other, repeatedly.

(11) Later: Hand bent in 90º to the left; index finger straight out, moving in a circular way.

(12) Hitch hiking: Arm bent in 90º; hand closed, thumb out. Arm repeatedly moving away from the body to the right side.
